# Exploring Oral Health in Children From Culturally and Linguistically Diverse Backgrounds Using Social Practice Theory Lens: A Scoping Review

**DOI:** 10.1111/cdoe.70011

**Published:** 2025-08-17

**Authors:** Rashmi Jamkar, Paul R. Ward, Hanny Calache, Colleen Fisher, Virginia Dickson‐Swift, Ivana Matic Girard, Linda Slack‐Smith

**Affiliations:** ^1^ School of Population and Global Health The University of Western Australia Perth WA Australia; ^2^ Research Centre for Public Health, Equity and Human Flourishing Torrens University Adelaide South Australia Australia; ^3^ Department of Rural Clinical Sciences, La Trobe Rural Health School La Trobe University Bendigo Victoria Australia; ^4^ Violet Vines Marshman Centre for Rural Health Research La Trobe University Bendigo Victoria Australia

**Keywords:** children, culturally and linguistically diverse, high‐income countries, oral health, social practice

## Abstract

**Objectives:**

There is evidence that children from culturally and linguistically diverse (CALD) backgrounds in high‐income countries experience a higher burden of oral diseases compared to children from non‐CALD backgrounds. Oral disease remains a significant health problem in high‐income countries, and the success of current traditional approaches to manage oral diseases has been limited. Thus, it is time to examine other approaches that look beyond the individual and focus on the wide‐ranging influences, including context. One such approach is the use of social practice theory (SPT) which examines the 'practice' (an everyday activity), how it happens, and what is required to engage with it. This review aimed to map out oral health‐related practices across international literature through the three elements of the SPT framework (materials, meanings and competences) in children from CALD backgrounds in high‐income countries.

**Methods:**

This scoping review followed Joanna Briggs Institute's Population, Concept and Context framework. MEDLINE database was initially searched via a librarian guided search strategy to retrieve relevant studies. The words from titles and abstracts from relevant studies and index terms were later used to develop a full search strategy, which was then used to search Scopus, EMBASE, MEDLINE, PsychINFO, CINAHL, Public Health Database and Dentistry and Oral Sciences Source. The reference lists from all retrieved studies were screened for any additional relevant studies. Peer‐reviewed qualitative and quantitative, mixed‐methods and systematic review studies published in English were included. Screening of eligible studies and data extraction was performed in Covidence. Data extracted from each study was analysed and interpreted using Shove's SPT framework.

**Results:**

Thirty‐seven studies were included in the review. A number of key oral health‐related social practices such as feeding children, sleeping, using a comforter, teeth cleaning and health and care oriented mobility were identified in children from CALD backgrounds along with their three elements: materials, meanings and competences.

**Conclusion:**

Using a SPT lens allowed a new way of exploring family, cultural and community factors and moving away from the restrictive focus on individual behaviour. Focusing future research on these dynamics of practices can provide insights into the impact of barriers and facilitators on their implementation of interventions and identify opportunities for leveraging positive change.

## 1 Introduction

### 1.1 Background

Oral diseases in children are a major burden, particularly dental caries (tooth decay) and periodontal diseases (gum problems) [1, 2]. Untreated oral diseases can cause physical symptoms, functional limitations (speaking, eating, and breathing) and impact on emotional and social well‐being [1, 2]. Research in oral health of young children from CALD backgrounds in high‐income countries suggests that CALD children are disproportionately affected compared to children from non‐CALD backgrounds [3, 4, 5, 6, 7, 8, 9, 10, 11].

This review includes studies focused on CALD children from high‐income countries [12]. Commonly used in Australia, CALD may be referred to by different terms in other high‐income countries to describe similar populations. CALD in this study refers to people residing in host countries who were born outside the host country or are descendants of those born out of the host country and differ in a language and/or culture to that of the wider population [13, 14]. This definition does not include First Nations people [14]. Research focusing on the oral health of CALD communities suggests that poor oral health in CALD children may be associated with factors such as country of origin, diet, acculturation, traditions, habits, and customs [5, 15, 16, 17, 18]. Structural determinants of health may also have an important impact on oral health as they encompass proximal (e.g., access to services) and distal (e.g., economic policies) factors [19]. Recent research supports the notion that children's oral health, including oral health practices, eating habits, and utilisation of dental services by CALD communities, are all influenced by cultural factors beyond biology and diet. These factors can affect oral health outcomes through dietary practices influencing oral health, and acculturation affecting access to oral healthcare [15, 16, 18, 20, 21, 22, 23]. Culture is not limited to religion but includes values, attitudes, beliefs, habits, and customs and any other competences learned by an individual since the beginning of childhood but is not necessarily taught [24]. While the terminologies differ, the experiences of these population groups, particularly related to health care, are often shaped by common challenges such as language barriers, cultural beliefs, and structural determinants of health [25].

The aim of the review was to elicit social practices related to oral health that emerge from international literature through the three elements of SPT lens in children from CALD backgrounds. While studies rarely explore social practices per se, by examining day‐to‐day activities in families through knowledge, attitudes, and practices, underlying practices may be uncovered implicit in such research. The practices between children from CALD and non‐CALD backgrounds may not necessarily be different but they may be performed differently in terms of materials, meanings and competences. An example: while teeth cleaning is common practice in both CALD and non‐CALD groups, some people from CALD backgrounds prefer to use traditional aids as materials to clean their teeth instead of conventional toothbrush and paste because of their cultural beliefs that traditional aids are more effective [26]. This review focuses on high‐income countries because studies conducted in high‐income countries show similar reports concerning oral health in CALD children [3, 4, 5, 6, 7, 8, 9, 10, 11] and have similar economies. Hence, they are more comparable.

Current research in oral health remains focused on individuals' behaviour, prevention, and treatment [27]. Clinical procedures contribute minimally to improve oral health and cannot eradicate oral diseases alone [28, 29]. Individualistic and behavioural change theories often neglect social conditions, context, and complexities of everyday life [30, 31, 32, 33]. All of the above ignore social and contextual factors behind poor oral health. They often overlook daily routines and practices that shape everyday life [29, 34, 35, 36, 37]. Conversely, theories of practices emphasise everyday routines and practices [38]. SPT has gained popularity in health research, particularly in studying alcohol consumption, smoking, obesity and oral health‐related practices [37, 39, 40, 41, 42, 43]. Recent research using SPT in oral health takes the focus away from individual behaviours to the social context in which children's oral health is embedded [42]. Identifying different elements and how they link with each other for a practice to be performed helps understand their broader influence on oral health [32].

### 1.2 Social Practice Theory: A Brief Overview

Different theorists including Giddens, Bourdieu, Reckwitz, Schatzki, Shove and Hui share the belief that ‘practice’ is central to social life [44, 45, 46, 47, 48, 49]. SPT theory suggests that for understanding and analysing everyday lives, routines, and behaviours one needs to focus on the ‘social practice’. Reckwitz defines practice as “a routinised type of behaviour, however not limited to behaviour, but includes elements such as mental activities, bodily activities and individuals performing the activity” [46].

**FIGURE 1:**
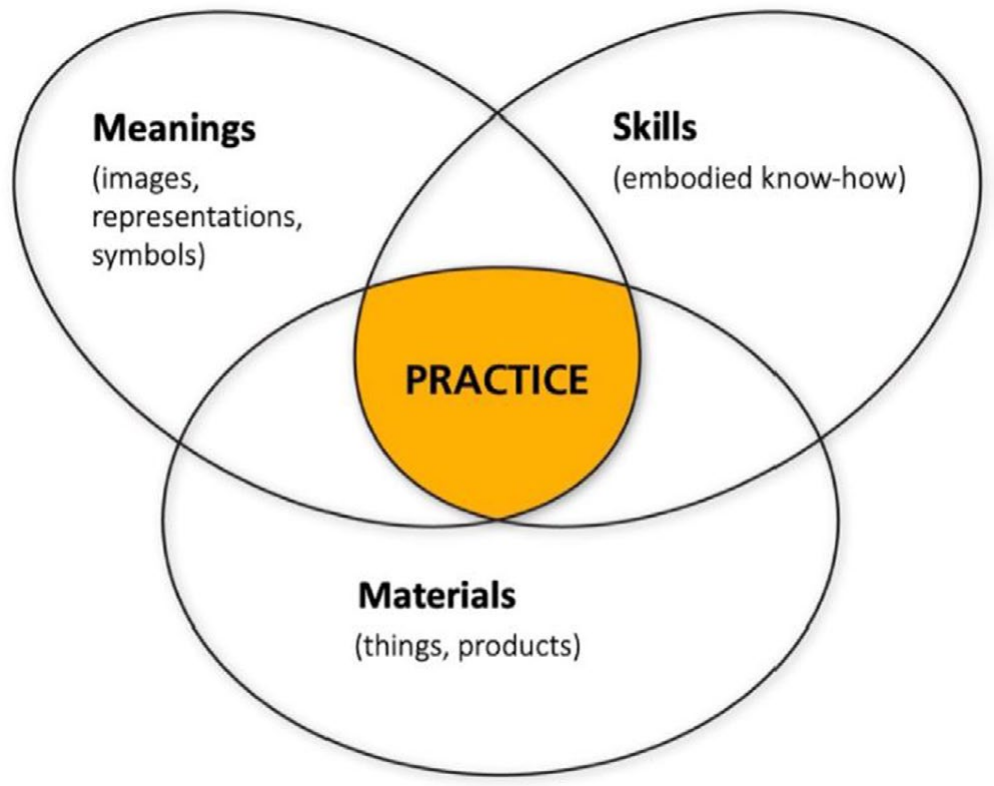
Shove et al.'s social practice theory framework [48].

Shove et al. describe social practice as comprising of three elements: *Materials*: objects, infrastructures, physical surroundings. The materials alone have no value and can lie dormant for years unless they are integrated in a practice by practitioners [50], *Meanings*: social and symbolic significance, purpose of enacting a practice. It shapes the perceptions, preferences, actions and interpretation of the social world [51] and *Competences*: knowledge, skills, ‘practical understandings’ to perform the practice [48] (Figure 1) [52]. For a practice to be performed all three elements must exist and be interconnected. SPT also emphasises that practices are not static but are enacted and evolved across time and space that is, the temporal and spatial aspect of practices [48]. The temporal aspect refers to the ‘time’, duration of a practice, unfolding of a practice, and the spatial aspect refers to ‘space’, physical location, and geographical context of a practice [48]. A practice can be influenced by geographical context which shapes the availability of resources to perform a practice. The persistence/cessation of practices depends on the interconnectedness of both the three elements and different practices [46, 48]. By identifying these elements, service providers can create/target interventions to address the needs and challenges within communities which can, in turn, lead to improved oral health.

### 1.3 Research Objectives

1. To map knowledge, attitudes and practices from existing literature on oral health in children from CALD backgrounds.
2. To use literature identified from objective 1 to identify key oral health related social practices in children from CALD backgrounds.

## 2 Methods

A scoping review was undertaken because of its broader net compared to systematic review, less restrictive inclusion criteria and relevance for exploratory research [53]. This scoping review and review protocol was guided by Joanna Briggs Institute's (JBI) framework for scoping reviews [53]. The research team comprises of individuals with various backgrounds including CALD (India, Egypt and Serbia).

### 2.1 Eligibility Criteria

The eligibility criteria for this review was established using the Population (or participants)/Concept/Context (PCC) framework by JBI [53] and is described in Table 1.

### 2.2 Search Strategy

To ensure comprehensive search of the literature, the initial search strategy was developed with the librarian using Boolean operators ‘AND’ ‘OR’ and combining four terms: ‘culturally and linguistically diverse,’ ‘oral health’,’ young children families' and ‘practice/attitudes/knowledge’ and related synonyms with a complete list detailed in Table S2. MEDLINE (via Ovid) database was initially searched, to retrieve relevant studies. The words from titles and abstracts from relevant studies and index terms were later used to develop a full search strategy which was then used to search Scopus, EMBASE (Ovid), MEDLINE (via Ovid), PsychINFO (Ovid) and CINAHL (via EBSCO host), Public Health Database (ProQuest) and Dentistry and Oral Sciences Source [DOSS] (via EBSCO host). A range of synonyms were used for ‘culturally and linguistically diverse’ to ensure a comprehensive search, as authors may have used alternative terms when describing similar concepts. CALD is not a familiar term across international literature hence synonyms such as migrants, immigrants, refugees, asylum seekers, ethnic groups, transients, migrant families, immigration and emigration were included in the search strategy across all seven databases. A systematic review conducted among CALD groups in Australia successfully used similar synonyms to assess relevant literature [54]. The reference lists from all retrieved studies were screened for any additional relevant studies. The search was conducted in December 2023.

Citations retained after screening were uploaded to the citation manager EndNote 21 [55] and duplicates removed. The citations were then uploaded to Covidence [56] where a second duplication check occurred. Two reviewers screened titles and abstracts, then conducted a full text review of the included studies. Any reviewer disagreements were resolved through discussions. Screening of studies and data extraction was performed in Covidence.

### 2.3 Data Charting Process

Data were extracted in two tables (Tables S3 and S4). One included study (authors and year), aim, setting, sample size, participant characteristics, study design, language used for data collection, and knowledge, attitudes and practices. The second table focused on social practices and was structured as three elements of practice (materials, meanings and competences) along with temporal and spatial aspects.

## 3 Data Analysis

Data were extracted based on key characteristics of the studies. A combination of inductive and deductive approaches was used to extract data related to the identified social practices in the included studies [57]. Data extraction focused on information that explored any activities related to oral health in children for example, feeding, toothbrushing. The final 37 studies were coded using NVivo software. An inductive approach was used to review the studies to develop codes for the extracted data. A deductive approach was used to assign appropriate codes to categories of SPT (materials, meanings, competences, temporal and spatial) by the lead investigator (RJ) in consultation with the co‐authors.

## 4 Results

**FIGURE 2:**
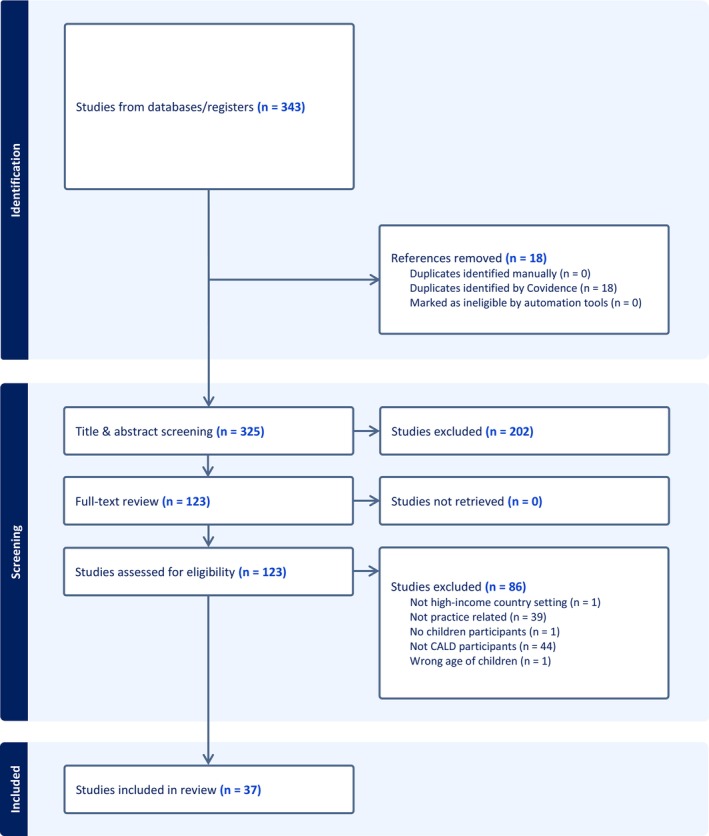
PRISMA flowchart.

A detailed PRISMA flowchart (Figure 2) describes the assessment and exclusion of studies. Overall, 343 studies were identified after which 18 duplicates were removed. Initial screening excluded 202 studies. Out of the remaining 123 studies, 86 were excluded based on eligibility criteria including: not a high‐income country setting (*n* = 1), studies not suitable for exploring practices (*n* = 39), no child participants (*n* = 1), no participants from CALD backgrounds (*n* = 44) and irrelevant child population (*n* = 1). The final review included 37 studies. The review results do not include findings from non‐CALD background participants.

The studies were published between 1982 and 2020. The included studies came from seven high‐income countries: the United States (*n* = 14), the United Kingdom (*n* = 5), Norway (*n* = 5), Australia (*n* = 4), Sweden (*n* = 4), Canada (*n* = 3) and the Netherlands (*n* = 2). Out of 37 studies, participant groups varied across studies; 17 included parent/mother/caregivers/families‐child dyads, 16 included mother/parents/caregivers/families only; one included mothers, grandmothers; one included paediatricians, mothers; one included only children, and one included key informants, caregivers. The most used approaches for data collection were interviews (*n* = 18) (semi‐structured/phone based/open and closed‐ended questions) followed by surveys (*n* = 10), focus groups (*n* = 7), questionnaires (*n* = 8) and clinical examinations (*n* = 5). Two studies employed observations with interviews; one study utilised case histories, and one used discussion groups. Table S3 available in the Supporting Information document outlines the characteristics of the included studies.

## 5 Synthesis of Results

### 5.1 Knowledge, Attitudes and Practices

A total of 10 of 37 studies addressed knowledge of participants relating to oral health such as knowledge about looking after teeth: importance of primary teeth [23], understanding the importance of tooth brushing [58, 59, 60, 61, 62], looking for oral health related signs such as stains on the tooth or crumbling tooth structure in children [63, 64]; knowledge about what to feed children: impact of sugary drinks and foods (diet) on children's oral health [65], breastfeeding and bottle feeding impact on teeth [66], knowledge of traditional remedies for dental problems [67], awareness of socio‐economic factors on oral health practices and access to dental care [68, 69, 70].

Fifteen of 37 studies addressed attitudes of participants: attitudes regarding looking after teeth: giving little importance to primary teeth [23, 67, 68], irregular visits to dentists or waiting to visit the dentist only after a dental problem arises [23, 64, 71, 72], negative/less favourable attitudes towards dental care influenced by cultural beliefs [18, 73, 74, 75]; parental fear in relation to dental treatment [18], attitudes relating to feeding: using food as a reward/comfort for children [65, 76], and mistrust in advice from health professionals [77].

A total of 28 out of 37 studies addressed practices impacting children's oral health such as practices around feeding children: regular breastfeeding [11, 66, 77, 78, 79, 80, 81, 82], bottle‐feeding, night bottle feeding [11, 18, 61, 65, 66, 77, 80, 81, 83] adding sugar to the drinks [61, 77, 80, 83, 84, 85], premastication of food [67, 68], and frequent or ad‐libitum snacking on sugary drinks/foods [11, 65, 74, 75, 77, 82, 86, 87]. Practices related to oral hygiene included: regular toothbrushing [18, 58, 59, 61, 74, 75, 77, 79, 86, 88], teeth cleaning [85] brushing after meals [58] and using traditional/home remedies for dental problems [67, 89].

### 5.2 Oral Health Related Social Practices

The aim of the review was to explore the literature to elicit any oral health‐related social practices in children from CALD backgrounds. Different practices get grouped together, and the elements connecting them form bundles or complexes, which illustrate how different practices are linked and how their elements work together [38, 43]. A number of social practices were identified across the included studies. Feeding children and health‐and care‐oriented mobility are two of the identified social practices which are discussed as broader social practices, and they consist of constellations of practices, as explained below. The other practices: using a comforter, sleeping, and teeth cleaning are discussed as discreet social practices. For the ease of the reader, participants from studies will be referred to as caregivers unless specified otherwise. All the elements for each practice below are not listed but only key findings from the included studies. A detailed list of elements for each practice (broad and narrower) is contained in Table S4.

### 5.3 Feeding Children

**FIGURE 3:**
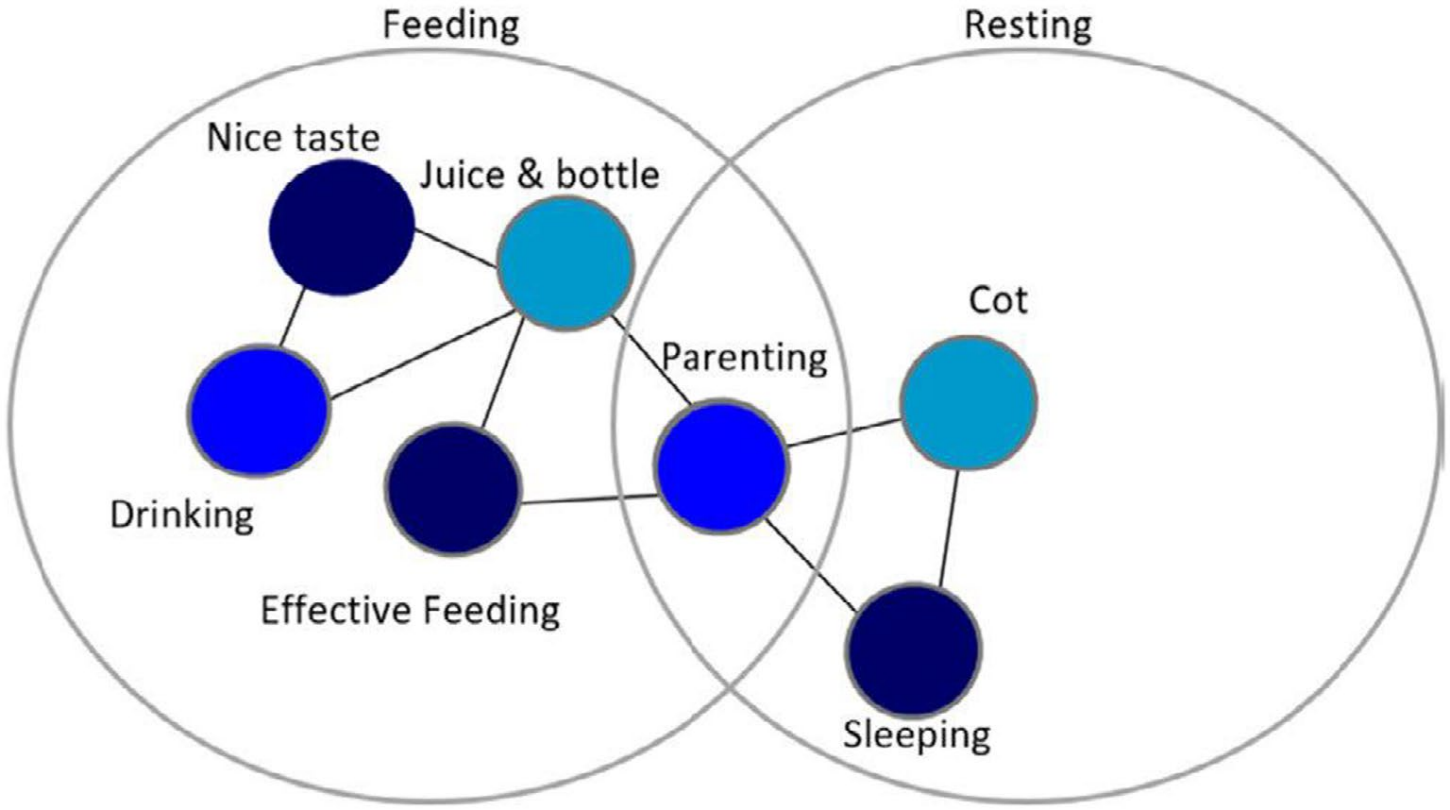
Practice bundle [43]. This is a representation of different practices linking with each other due to various elements and forming a practice bundle.

The analysis identified breastfeeding, bottle feeding, and feeding foods as three inter‐related yet distinct practices within the feeding practice bundle [33, 43]. While these practices are collectively oriented towards child nourishment, they are distinct through their unique elements. Each practice has unique physical resources (e.g., snacks, bottle, utensils), culturally embedded meanings (e.g., maternal anxiety, social expectations) and embodied competences (e.g., adding sugar to drinks in bottles). The bundling reflects the overarching aim of feeding but highlights how feeding is not a single homogenous activity; rather, it is enacted through distinct socially situated practices such as bottle feeding, breastfeeding, and sleeping. An example of a feeding bundle from a study is attached for understanding (Figure 3).

#### 5.3.1 Feeding Foods Practice

A variety of foods and drinks were mentioned in the included studies to aid in feeding. Solid foods such as sugar and salt‐containing processed foods were identified as commonly used in feeding [58, 64, 78, 82, 84, 85, 88, 90]. Studies documented the influence of cultural, family routines and parental beliefs: maternal anxiety, social expectations, that led the caregivers to feed foods that are harmful to children's oral health [82, 84, 90]. Competences reported from studies suggested caregivers learning strategies such as distracting the child using television or toys when feeding, prechewing baby's food prior to feeding them, and modifying the children's food taste as per their (caregivers) own reflecting how competences are shaped by cultural expectations, prior parenting experience and traditions [11, 67, 76, 84, 89, 90]. Studies reported using snacks (material) for feeding; snack was a ‘reward’ for good behaviour, fairness in family to give sugary snacks, and accepting sugary snacks from extended family depicting social, cultural and emotional meanings behind feeding sugary snacks [58, 75]. In spite of awareness of the effects of sugar on teeth, maternal need for child to be eating often drove the practice of feeding towards consumption of drinks/foods high in sugar.

#### 5.3.2 Breastfeeding Practice

Included studies reported breastfeeding practised among the caregivers primarily organised around the use of maternal body (material). One study reported how a cultural group practiced breastfeeding not because it was good for the baby but because it was a way of birth control in their culture. “If you want to space you kids, you have to breastfeed.” (caregiver)^82(p310)^ Other meanings identified were it being economical, antenatal advice provided by health professionals, and emotional such as parental preference [80, 82]. One study observed breastfeeding was practiced in low proportion in Asian mothers compared to other groups [80]. Competences explicit to breastfeeding were not observed among the studies but reading cues for child's hunger, providing breast when the child wakes up in the middle of the sleep were evident in studies [61].

#### 5.3.3 Bottle‐Feeding Practice

This practice was characterised by the use of physical objects such as bottles, milk, milk products, sweet‐tasting drinks, and bottle nipples across several studies [18, 58, 61, 64, 67, 71, 77, 80, 82, 84, 88, 89, 90]. Meanings identified for bottle‐feeding were varied; for example, children were satisfied with the bottle and mothers were happy providing a bottle in spite of being aware that drinks in the bottle were linked with endangering teeth [82]. Working caregivers (mothers) preferred bottle‐feeding as it was practical [71] some caregivers performed bottle‐feeding to encourage milk consumption and because doctors advised [82]. Other key meanings for using bottle‐feeding included unawareness of which solid foods to start, the cultural need for the food to be halal, and concerns over water safety from the birth country prioritising sweetened drinks over water [67, 77]. One study with paediatricians mentioned (meaning) how parents were obsessed with feeding children and hence used bottles to force‐feed [90]. Competences related to bottle‐feeding included knowing to cut larger holes in bottle nipples to feed a large amount of semi‐solid foods/drinks [90], to add sugar to sweeten the bottled drinks to encourage more consumption fuelled by the maternal need for more consumption [82].

Several studies provided evidence for the temporal aspect of feeding; a pattern of prolonged bottle feeding was observed (15 months to 4 years) [11, 61, 71, 78, 80, 82, 83, 89]. It is worth noting that even though some caregivers were aware of when to stop bottle feeding, it did not occur, which also delayed introducing solid foods in children [90]. Temporally, the consumption of snacks and frequency varied [75, 78, 84], with one study mentioning how children snacked often ad libitum throughout the day [65]. Spatially, it was observed that the consumption of unhealthy food/drinks depended on proximity and availability of stores selling sweet drinks [67], being at a relative's house where usually children were provided with unhealthy snacks [82]. Being in refugee camps and newly moving to host countries also impacted what caregivers shopped for, which subsequently impacted feeding [61, 64, 67].

### 5.4 Using a Comforter

A comforter is commonly used to provide comfort to children in distress and promote better sleep. This practice was identified relating to oral health because materials mentioned in some studies suggested use of foods high in sugar for comforting, such as bottles filled with sugar‐containing liquids (materials) [61, 84]. One study reported that although mothers were aware of the negative effects of sugary foods on their children's dental health, they still used sweetened comforters to soothe distressed children, prioritising emotional comfort over potential harm (meaning) [84]. The same study also reported that as the number of teaspoons of sugar mothers used increased, so did their tendency to add more sweetening agents to their children's comforters, indicating strong emotional preference (meaning) despite being aware of sugar's negative effects on teeth [84].

On the temporal aspect, two studies reported how using comforters ranged from being used at night to sometimes the whole day [78, 84].

### 5.5 Sleeping

Sleeping at first glance might seem unrelated to oral health, but it is a social practice embedded in routines and interlinked elements with other practices such as night teeth cleaning and night feeding (Figure 3). Several studies in the review reported using sugary drinks filled bottles [18, 61, 69, 82], and sweetened pacifiers [67, 81, 82] (materials) to aid in sleeping which suggests a direct impact on oral health. Sleeping was associated with cultural meanings: good sleep leads to good health (meaning) for which they would often provide sugary drinks in bottles for sleeping [58]. Another important meaning identified was social expectations, preventing disturbance to other household members. Hence a child would have been put to sleep using sugary drinks [71]. Competences observed related to sleeping were conflicted as despite caregivers being aware of sugary foods being harmful for teeth, they added sugar/sugary drinks to the bottle/comforters [58, 67, 69, 82]. One significant finding from a study was the ‘bottle propping’ which even though is not a universal practice was commonly practiced across all population groups which again can aid in providing sugar‐rich drinks to children throughout the night [82] in turn impacting children's oral health.

Temporally, one study mentioned children were often given sugar‐containing liquids during sleep at the age of one [78]. One study identified that caregivers of varied CALD backgrounds commonly provided their children with bottle/breast whenever the child woke up, and some continued to give the bottle all night long [82]. Spatially, it was identified that materials added to a bottle were different, with participants continuing to utilise knowledge gained from their home countries [67]. Children's sleep prioritised over other oral health practices such as brushing or avoiding sugary drinks to sleep suggests how sleeping with bottles/comforters is hard to change because it is closely associated with cultural values and socially expected caregiving routines despite caregivers' awareness of the effects of sugar on teeth.

### 5.6 Teeth Cleaning

The review identified teeth cleaning as one of the social practices from the included studies which was regularly performed by the CALD communities using a combination of different materials. Materials identified included conventional child‐centric materials: toothbrush, toothpaste, fluoridated toothpaste, as well as traditional materials [58, 67, 69, 89, 91] although it was not evident from the studies if the caregivers used traditional cleaning materials to clean their and/or their children's teeth. Two contrasting findings were reported in two studies on use of materials: in one where caregivers used traditional objects to clean their and/or their children's teeth due to cultural beliefs of traditional objects being more effective [67] while the other one reported caregivers using fluoridated toothpaste because they knew the importance of using fluoridated toothpaste [69]. This suggests caregivers relied on their cultural and personal knowledge when it came to teeth cleaning. The most common meaning for teeth cleaning was preventing tooth decay and removal of bacteria [58, 60, 66, 75, 86]. Another key meaning identified was children wanting to copy their parents' brushing habits [58]. These meanings provide teeth cleaning with cultural and social value. Competences relating to teeth cleaning were shaped by parental knowledge (importance of primary teeth, how to brush [58, 86], using fluoridated toothpaste) [69], and intergenerational teaching (using traditional objects) [67]. Some caregivers used different strategies such as buying character brushes which they knew their children would like or making a game out of brushing so children would not think of it as a task and would enjoy cleaning [58].

Children were taught to brush their teeth from a very young age, suggesting a temporal aspect [86]. Spatially, day care centres were identified as a place where children were taught to brush daily [86]. The findings highlight how teeth cleaning is a collective routine rather than an individual choice.

### 5.7 Health and Care‐Oriented Mobility Practice Bundle

This review identified practices such as attending a dental clinic, attending a health centre, and attending prenatal classes across the included studies as contributing to the health and care‐oriented mobility practice bundle. Understanding this practice bundle was important as it directly and indirectly highlights how distinct practices mentioned above can support early engagement with oral health services and, therefore, impact oral health in children. This bundle is described as a mobility practice bundle because it highlights the movement of caregivers across different spaces (social, healthcare). It also exemplifies how caregivers carry across different elements as they traverse different settings.

For the practices of attending centres (health/dental) mentions of materials such as health centre, dental clinics, and transportation resources (e.g., car) were reported [64]. Meanings associated with these practices varied, with preventing dental pain being the most common [63, 71, 72, 82, 90]. Other meanings were cultural beliefs, societal expectations [67, 86] avoiding low self‐esteem [58, 63] and negative dental experiences of family members [58]. Health professionals played a pivotal role in introducing visiting dental/health centres for children's health and nutrition (competences) [23, 64, 80]. Similarly, attending prenatal classes (Lamaze) equipped caregivers with knowledge about taking their children to the dentist [82]. Other competences observed in three studies were scheduling dental appointments and awareness of visiting dental clinics [68, 89, 90].

Temporally, children's dental visits varied, ranging from 2–3 years to the child being in elementary school [64, 68]. Spatially, issues identified across practices were rural clinic locations and waiting to visit the caregiver's home country to receive the necessary treatment [71, 87].

## 6 Discussion

This scoping review identified various social practices (broad and narrower) related to oral health in children from CALD backgrounds: feeding practice bundle, using a comforter, sleeping, teeth cleaning, and health and care oriented mobility practice bundle. The three elements (materials, meanings and competences) of the identified practices can be regarded either as resources that enable or restrict certain practices that impact oral health in these children.

Shove et al.'s SPT framework, was applied to study the elements identified in the studies included to elicit oral health‐related practices in children from CALD backgrounds [50, 92]. This was achieved through deconstructing a practice and an analysis of the elements (materials, meanings, competences) that enable the practice to be performed. This review is not without limitations. SPT is an abstract theory which can be challenging to interpret and apply [93, 94]. It leaves room for subjective interpretation, and interpretations can differ from researcher to researcher [92]. The search strategy used may have had limitations, such as language restrictions. This review only included studies conducted in high‐income countries, making their applicability to low‐ and middle‐income countries uncertain. Additionally, it did not include an assessment of the quality of the included studies. Although this review did not focus on examining structural determinants of health, their influence, however, cannot be ignored. Factors such as socioeconomic conditions, accessibility to services, policies, and language barriers are likely to shape oral health‐related social practices and should be considered in future research.

Various materials identified across the practices reveal how a variety of objects were instrumental in achieving a practice ranging from traditional to modern day objects. SPT considers material as an active element as it shapes and influences how a practice is carried out, sustained, or ceased [48]. Similar to these findings, and unique to people from CALD backgrounds, previous research also identifies the use of traditional materials for oral health care in CALD backgrounds participants [95] with these traditional oral hygiene practices being retained when participants moved to settlement countries [91].

Different meanings were identified across the practices. The meanings revealed various cultural and emotional reasons for performing certain practices. One meaning identified was family demographics: lone parents used more sugar in their own tea/coffee compared to married participants, which led them to do the same when providing drinks to their children [84]. This might suggest that parents' own preference for sweet taste can influence the sugar content in their children's diet, impacting children's oral health. Cultural norms/inappropriateness were also identified as meanings for feeding sugary foods/drinks, suggesting focusing on the cultural factors that can influence oral health‐related practices. Identified meanings behind oral health‐related practices suggest the saliency of focusing on family demographics, deeply rooted cultural norms, environmental and economical contexts when researching oral health in children. Using SPT as a framework would bring these contexts to light.

Included studies provided limited information around competences related to the identified practices. There were, however, studies that demonstrated how parents acquired necessary skills to perform certain oral health‐related practices [11, 67, 90]. Having knowledge of using traditional materials for teeth cleaning demonstrated deeply rooted cultural knowledge [67, 69, 89, 91]. Findings also highlighted how personal networks can be an important factor that may influence oral health‐related practices in children from CALD backgrounds [82]. There is evidence in the literature that community networks have a long‐term effect on the oral health quality of children [96]. There was limited focus in the included studies of this review on how and where caregivers gathered their knowledge around oral hygiene practices, which is a cue to use SPT in future research to understand where and how knowledge has been acquired, for example, passed down from generations or acquired from health professionals, personal or community networks.

There was limited data on the temporal aspect of practices in the included studies. Findings from one study [58] reported how caregivers prioritised attending a party over the child's dental appointment, which suggests how practices compete for time [48]. Further research into focusing on the temporal aspect of practices is important because temporality will help understand which practices take priority and which oral health‐related practices do not occur because of competing demands.

There was limited data around the spatial aspects of practices. It is possible that the researchers' focus was not on the geographical context, physical space, or locations, resulting in the spatial concept not being explored. Spatial data identified for certain practices revealed how the geographical locations can be crucial to their performance. For example, day care centres introduced the practice of brushing in children [86]; attending a Lamaze class introduced the practice of visiting a dental clinic for some caregivers [82]. Knowing the spatial aspects of social practices is important as it enables an understanding of how a practice changes/diffuses and elements modify as the location changes [48] which can either be beneficial/harmful to oral health in children.

The findings of the review have implications for public health policy and practice. Understanding oral health in terms of social practices provides a more holistic understanding than examining individual behaviours, which has traditionally been the case. The subsequent interventions and supports will, therefore, be more likely to account for social context in their development and implementation. Using SPT to further study oral health‐related traditional practices and materials that intertwine with human action and the social order is required to study the impact of such interventions [38]. The findings from this review can be used for subsequent studies for comparison between children from CALD and non‐CALD backgrounds, which can tell us if the practices are unique/different and if so, what elements of those practices make it different between the groups.

## Ethics Statement

This review does not require ethics approval since this scoping review will examine de‐identified data from publicly available materials and does not involve human participants [97].

## Conflicts of Interest

The authors declare no conflicts of interest.

## Supporting information


**Tables S1–S4.** cdoe70011‐sup‐0001‐Tables.docx.

## Data Availability

Data sharing is not applicable to this article as no new data were created or analyzed in this study.
