# Supplementary material for: Exploring Oral Health in Children From Culturally and Linguistically Diverse Backgrounds Using Social Practice Theory Lens: A Scoping Review
Source: Community Dent Oral Epidemiol. 2025 Aug 17;53(6):644–54. doi: 10.1111/cdoe.70011 (PMC12627272; doi:10.1111/cdoe.70011)
Supplement: Supplementary file 1 — Tables S1–S4. cdoe70011‐sup‐0001‐Tables.docx. [file CDOE-53-644-s001.docx]

SUPPLEMENARY MATERIALS

**Table 1: Inclusion and exclusion criteria**

| **Inclusion criteria** | **Exclusion criteria** |
| --- | --- |
| **Type of publication**: Peer-reviewed qualitative, quantitative, mixed-methods studies or systematic reviews.  **Timeframe**: No limit was placed to the date of publication.  **Language**: Only studies published in English language.  **Population**: Families/caregivers with at least one child aged zero to five years from culturally and linguistically diverse backgrounds.  **Concept**: Studies were included if words such as practice/knowledge/attitudes & related synonyms concerning oral health were mentioned.  **Context**: studies conducted in high-income countries ^1^ (studies conducted in high-income countries showed similar reports concerning oral health in CALD children and have similar economies hence they are more comparable) | Studies which do not include culturally and linguistically diverse population. Studies including families/caregivers of children older than 5 years of age. Studies that focused only on First Nations participants.  Studies which do not mention families’ accounts of oral health related behaviours/practices/knowledge/attitudes.  Studies conducted in low-income countries. |

**Table 2: Complete list of search terms used across all databases**

|  | **CONCEPT 1**  AND | **CONCEPT 2**  AND | **CONCEPT 3**  AND | **CONCEPT 4** |
| --- | --- | --- | --- | --- |
| **KEY TERMS**  **OR** | Culturally and linguistically diverse | Pre-school children | Social practices | Oral health related |
| **SYNONYMS/ ALTERNATE TERMS** | Migrants, immigrants, refugees, asylum seekers, ethnic  **OR** | Toddlers, very young children, babies*, parents of young ones, care givers | Everyday activities, behaviour, routinized activities | Toothbrushing, feeding, |
| **Medline MeSH Subject Headings** | Refugees/  Transients and migrants/  migrant families, ethnic groups, immigration, emigration | Child/  Child, preschool/  Parent-child, | Health behaviour/  Attitude to health/  Knowledge, parental attitudes, feeding behaviour | Toothbrushing/  Dental caries, food habits,  Oral health |
| **Embase Emtree Subject Headings** | Refugee/Migration, immigration, refugees | Preschool child/ | Mother’s knowledge, practices and beliefs of parents, feeding behaviour, psychological aspect, self-concept | Tooth brushing/  caries |

**Table 3. Characteristics of the included studies**

| **Study** | **Aim** | **Settings** | **Sample size** | **Participant characteristics** | **Study design** | **Language for data collection** | **Knowledge (1), Attitudes (2) & Practices (3)** |
| --- | --- | --- | --- | --- | --- | --- | --- |
| Dykes et al. 2001 ^2^ | “To assess the role of socio-economic factors in explaining ethnic differences in two infant feeding practices contra-indicated for dental health: adding sugary foods to a feeding bottle and bottle usage for any type of drink after one year.” | United Kingdom | 2598 Immigrant & 548 White families | Bangladeshi, Pakistani, Indian, White | Survey, Face to Face interviews | Not mentioned | - Providing sugary drinks to children (3) - Bottle-feeding up to 15 months of age (3) - Adding sugar to bottles (3) |
| Finlayson et al 2019 ^3^ | “To qualitatively explore psychosocial factors that could affect caring  for children’s oral health with a community-based convenience sample of low-income  Hispanic mothers of preschoolers on the US-Mexico border.” | United States | 36 mothers | Hispanic/  Mexican | Six focus groups and one individual interview | Spanish/English | - Importance of toothbrushing (1) - Providing sugary drinks to children (3) - Toothbrushing at the age of two (3) - Brushing after meals (3) - Visiting dentist regularly every six months (3) |
| Godson & Williams 1996 ^4^ | “To compare dental health and associated behavioural attributes among United Kingdom-born three-year-old children of first and second generation Pakistani Muslim mothers’ resident in areas of social deprivation in Bradford, West Yorkshire.” | United Kingdom | 226 children and their mothers | Pakistani | Home-based interviews with semi-structured questionnaires with both open and close ended questions | English/Urdu/  Punjabi | - Regular bottle feeding (3) - Using traditional remedies for oral hygiene (3) |
| Wong et al. 1996 ^5^ | “To determine the severity of nursing caries, and to examine contributing behavioural factors, in a group of Vietnamese families in British Columbia, Canada.” | Canada | 60 mother/child pairs | Vietnamese | Interviews | Vietnamese | - Importance of tooth brushing (1) - Prolonged bottle feeding (3) - Adding sugar to bottle (3) - Toothbrushing using toothpaste to clean children's teeth (3) |
| Hocking et al. 1982 ^6^ | “To provide information on infant feeding patterns.” | Australia | 437 children and their mothers | Australian, Middle Eastern, Southern European, Asians, United Kingdom, Others | Survey, Interviews | English | - Bottle feeding (3) - Prolonged bottle feeding (3) - Use of comforter for children (3) - Adding sugar to the bottle (3) - Children sleep with a bottle at night (3) |
| Hoeft et al. 2011 ^7^ | “To gain an understanding of urban Mexican American parents' knowledge, beliefs, and practices surrounding their young children's oral health, including first dental visit.” | United States | 48 caregivers (mothers) | Mexican | Qualitative Interviews | Spanish/English | - Awareness of dental visits (1) - Looking for oral health related signs on children’s teeth (1) |
| Contento et al. 1993 ^8^ | “To investigate the relationship between potential criteria mothers used to select foods for their children, their food knowledge, and food consumption of their children.” | United States | 218 mothers | Latinos, African American, Other ethnic backgrounds | Questionnaire | Spanish/English | - Using food as a reward for children (2) |
| Christian et al. 2015 ^9^ | “To explore dental service usage of 1–4  year olds from migrant families residing in metropolitan  Melbourne.” | Australia | 625 children | Iraqi, Lebanese, Pakistani | Self-reported questionnaire | English | - Not regularly visiting the dentist because they had no reason to visit the dentist (2) |
| Chomitz et al. 2019 ^10^ | “To describe the design, feasibility, and acceptability of a theory-informed  obesity and dental caries prevention pilot study, Baby Steps to Health, conducted in an  academic dental clinic among a primarily Asian immigrant population.” | United States | 50 caregivers on initial visit and 46 caregivers for the follow up phone interviews | Asian predominantly Chinese | Questionnaire, Phone interviews | English/  Cantonese/  Mandarin | - Using food as a reward for children (2) - Regular bottle-feeding at night (3) - Ad libitum snacking (3) - Frequent pressure by caregivers to children eat all the meals served (3) |
| Hilton et al. 2007 ^11^ | “To identify cultural beliefs, practices and experiences that influence access to preventive oral health care for young children from different racial and ethnic groups.” | United States | 177 carers of children | African American, Latino, Chinese, and Filipino groups | Focus groups | Spanish, Cantonese, Tagalog, English | - Less favourable attitudes towards preventive dental care influenced by community beliefs and personal negative experiences (2) - Visit to the dentist only if there was a dental problem (2) |
| Gibbs et al. 2016 ^12^ | “To describe early childhood caries in children from migrant families and explore possible influences.” | Australia | 630 children and their parents | Iraqi, Lebanese, Pakistani | Self-administered questionnaire | English | - Regular teeth cleaning (3) - Adding of sugar to the children's drinks/foods (3) |
| Barker & Horton 2008 ^13^ | “To examines the intersections among four societal sectors or contexts of care which  contribute to oral health disparities for low-income, preschool Latino children in rural California.” | United States | 30 Key informants, 47 caregivers | Mexican and Central American, first-generation Mexican Americans, Key informants | Semi-structured interviews, informal conversations, observations at various public places | Spanish/English | - Lack of knowledge on visible signs of dental caries (1) - Waiting to visit the dentist until a dental problem arises (2) |
| Karasz et al. 2014 ^14^ | “To understand the barriers and facilitators to caries prevention for young children of immigrant Bangladeshi families in New York.” | United States | 15 paediatricians, 13 mothers | Bangladeshi | Interviews with the paediatricians. Focus groups and in-depth interviews with mothers. | Bengali/English | - Delayed introduction of solid foods in children (3) - Washing their children's mouth with water in lieu of brushing (3) - Modifying nipples on the bottle to accommodate semi-solid foods (3) |
| Sherri 2010 ^15^ | “To establish baseline data about oral health knowledge, attitudes and behaviours of migrant and seasonal farm workers.” | United States | 45 parents | Mexican | Oral health knowledge, attitudes, and behaviours survey. Focus groups | Spanish/English | - Irregular visits to the dentist (2) - Prolonged bottle feeding (3) |
| Mustafa et al. 2020 ^16^ | “To assess toothbrushing-related perceptions among parents with immigrant background living in Norway.” | Norway | 233 parents | Asian, African | Face to face interviews | Norwegian/English/Other languages | - Awareness of breastfeeding and bottle-feeding impact on children’s oral health (1) - Regular breastfeeding in the night (3) |
| Nguyen et al. 2017 ^17^ | “To investigate the oral health knowledge, beliefs, and behaviours  of migrant Vietnamese parents of 1–5-year-olds in San Jose, California.” | United States | 45 parents | Vietnamese | Verbally administered survey | Vietnamese | - Awareness of socioeconomic factors on accessing oral health care (1) - Giving little importance to primary teeth in children (2) - Premastication of food before feeding the children (3) |
| Nicol et al. 2014 ^18^ | “To explore how humanitarian entrant refugees understand and make sense of good oral  health in pre-school aged children.” | Australia | 44 parents | Refugee participants from nine different countries - Myanmar, Iraq, Kuwait, Sudan, Afghanistan, Burundi, Democratic Republic of Congo, Rwanda, Nigeria | 8 Focus groups and 5 nurse interviews | Arabic/Karen/  English | - Awareness of traditional remedies for dental issues (1) - Giving no importance to primary teeth in children (2) - Premastication of food before feeding children (3) - Using traditional remedies for dental problems (3) |
| Watson et al. 1999 ^19^ | “To collect baseline data prior to initiating a community based,  oral health promotion program in an inner-city Latino community in Washington DC, populated by Central American immigrants.” | United States | children (n=142) and their parents (n=121) | Central American, South American, other countries | Survey | Spanish/English | - Awareness of socioeconomic factors on accessing oral health care (1) - Bottle feeding at night (3) |
| Weinstein et al. 1996 ^20^ | “To focus on the cultural and familial differences in feeding practices.” | United States | 1148 children and their mothers | Caucasian, African American, Hispanic, Native American, Asian, Others | Two questionnaires first when the child was six months of age and second when the child was twelve months of age. | English | - Awareness of socioeconomic factors on accessing oral health care (1) |
| Wendt & Birkhed 1995 ^21^ | “To describe oral hygiene factors in infants and toddlers living in Sweden with special reference to caries prevalence at 2 and 3 year of age and to immigrant status.” | Sweden | 632 children (original group) at one year (age), 593 children from original group were examined at two and three years (age) with their parents | Swedish & Immigrants | Clinical examination of the children and semi-structured interviews of the accompanying parents | Not mentioned | - Regular breastfeeding at night (3) - Using a comforter at night (3) |
| Stecksén-Blicks et al. 2004 ^22^ | “To examine caries and  some related background factors in 4-year-old children in Umeå in 2002 and to make comparisons with some of the findings from the earlier studies.” | Sweden | 218 children and their parents | immigrant children (9%) | Case histories | Not mentioned | - Awareness of importance of toothbrushing (1) - Toothbrushing at age of two (3) - Toothbrushing after meals (3) |
| Verrips et al. 1991 ^23^ | “To assess oral health of Turkish, Moroccan,  Surinamese, Dutch and "other" 5- yr-old children living in Amsterdam; second,  lo identify risk indicators for caries, in addition to ethnicity; and third,  to identify potential risk factors that are related to differences in caries experience in these children.” | Netherlands | 674 children, 525 parents | Turkish children (122) parents (107) Moroccan children (311) parents (258) Dutch children (94) parents (59)  Surinamese children (87) parents (56) Other children (60) parents (49) | Clinical examination, questionnaires | Turkish, Arabic, Dutch, Sranan Tongo, English | - Awareness of importance of toothbrushing (1) |
| Werneck et al. 2008 ^24^ | “To determine the influence of accessibility of dental services and other factors on the development of early childhood caries among Toronto children 48 months of age or younger with at least one Portuguese-speaking immigrant parent.” | Canada | 104 children and their parents | Portuguese speaking parents of children | Clinical examination & Interviews | Portuguese/  English | - Regular breastfeeding at night (3) - Regular bottle-feeding at night (3) - Using a sweetened comforter at night (3) - Ad-libitum snacking (3) |
| Wendt et al. 1994 ^25^ | “To describe oral  hygiene factors in infants and toddlers living in Sweden with special reference to caries prevalence at 2 and 3 yr of age and to immigrant status.” | Sweden | 532 Swedish & 61 immigrant children and their parents | A range of Immigrant backgrounds | Clinical investigation and interviews of accompanying parents | Not mentioned | - Regular breastfeeding at night (3) - Regular toothbrushing twice a day (3) |
| Williams & Fairpo 1988 ^26^ | “To enquire into toothbrush and toothpaste usage for infants by mothers resident in an inner-city area in the North of England, and to determine whether the oral hygiene behaviour reported varied between the different ethnic groups studied.” | United Kingdom | 175 Asian infants and a control group of 174 White infants and their families | Muslim (Pakistani (Mirpuri), Bangladeshi (Sylheti), Non-Muslim Sikhs from Punjab, Hindus from Gujarat of India, White | Interviews | English, Urdu, Punjabi, Hindi, Gujarati | - Using traditional materials to clean teeth (3) |
| Williams & Sahota 1990 ^27^ | “To determine attitudes of Muslim Asian mothers concerning infant feeding, weaning, the use of sugar and the cause of dental decay.” | United Kingdom | 100 mothers | Muslim Asians from the Sylhet region of Bangladesh and half from Mirpur in Pakistan. | Discussion groups | English, Urdu, Bengali, Punjabi | - Regular bottle-feeding (3) - Mistrust in advice from health professionals (2) - Regular breastfeeding (3) - Toothbrushing twice a day (3) - Frequent snacking (3) - Adding sweetening agents to children’s drinks (3) |
| Williams et al. 1989 ^28^ | “To determine the patterns of infant feeding established in an inner-city area within white and various Asian groups, and to consider the implications for health promotion initiatives.” | United Kingdom | Asian (n=184), White (n=127) families | Asian infants whose families originated from Mirpur in Pakistan, Sylhet in Bangladesh and Punjab or Gujarat in India (Sikhs and Hindus), Caucasian | Interviews | English, Urdu, Bengali, Punjabi, Gujarati, Hindi | - Regular breastfeeding (3) - Regular bottle-feeding (3) - Delayed introduction of solid foods (3) - Adding sweetening agents to children’s drinks (3) |
| Skeie et al. 2008 ^29^ | “To assess the relationship between parents’ dental attitudes and the caries increment in their children from the age of 3 to 5 years.” | Norway | 354 children when they were 3-year-olds from a previous study and 304 when they were 5-year-olds from same previous study and their parents | Norwegian and immigrants | Survey questionnaire | Not mentioned | - Negative attitudes towards dental care influenced by cultural beliefs (2) - Negative attitude towards diet associated with higher incidence of dental caries (2) |
| Skeie et al. 2010 ^30^ | “To investigate dental beliefs and attitudes of a diverse group of parents from their children when they were aged 3 and 5 years old and to identify possible mediators for a group composed of the parents with the most negative dental attitudes.” | Norway | 252 (3-year-old) and 345 (5-year-old) (these 5-year-old are from a previous cross-sectional study of 2002) and their parents | Norwegian group and immigrant group (Originating from Eastern Europe, Africa, Türkiye, South and Central America) | Questionnaires | Not mentioned | - Negative attitudes towards dental care influenced by cultural beliefs (2) - Regular toothbrushing (3) - Frequent snacking on sugary snacks/drinks (3) |
| Skeie et al. 2006 ^31^ | “To map existing disparities in oral health among immigrant and western native children in Oslo and to identify differences in parental, cultural and ethnic beliefs and attitudes towards oral health and caries-related behaviours.” | Norway | 770 western native & 130 immigrant children and their parents | Western native and immigrants. | Survey questionnaires | Not mentioned | - Less favourable attitudes towards dental care due to cultural beliefs (2) - Parental fear in relation to dental treatment (2) - Regular toothbrushing (3) - Regular bottle-feeding using sweetened drinks (3) |
| Skaret et al. 2008 ^32^ | “To explore the consistency of parental self-reporting of oral health habits, beliefs and attitudes towards child oral health care over a two-year period; 2) to evaluate possible differences in item scores and consistency between groups of parents with different immigrant status; and 3) to assess the construct validity of items measuring parental beliefs and attitudes toward child oral health care by exploring their relationship to the caries experience of the child.” | Norway | 273 parents of three-year-olds of western origin (western native) and 31 of non-western origin (immigrants) | Western native and immigrant groups | Questionnaire | Not mentioned | - Less favourable attitudes towards preventive dental care (2) - Negative attitude regarding diet and its relation to caries (2) - Regular toothbrushing once a day (3) - Frequent snacking on sugary snacks/drinks (3) |
| Shiboski et al. 2002 ^33^ | “To explore the role of race/ethnicity in the occurrence of early childhood caries among California Head Start (US) and non-HS preschool children.” | United States | 2520 children | Hispanic/Latinos, White, African American, Asian/Pacific Islander, Other/Multi-ethnic | Oral examinations, Questionnaires | Not mentioned | - Regular breastfeeding (3) - Using a sweetened comforter (3) - Regular bottle-feeding using sweetened drinks (3) |
| Rivera et al. 2020 ^34^ | “To explore Hispanic  seasonal farmworker caregivers’ beliefs and/or perceptions regarding early childhood caries their children’s oral health.” | United States | 20 parents/  caregivers | Hispanic/Mexican | Qualitative interviews | Spanish/English | - Regular toothbrushing (3) - Frequent snacking on sugary snacks (3) |
| Riedy et al. 2001 ^35^ | “To provide guidance for a public health intervention in a high  caries rate multicultural population by understanding cultural issues surrounding children’s oral health.” | United States | 41 women (mothers and grandmothers) | Chamorro, Filipino, Carolinian, Ponapean, Chuukese. | Focus groups | English | - Less favourable attitudes towards importance of primary teeth (2) - Regular breastfeeding (3) - Frequent snacking (3) |
| Perez & Amin 2014 ^36^ | “To define the underlying dimensions of psychosocial barriers to obtaining and providing dental  care for young children among recent immigrants.” | Canada | 99 primary caregivers/  parents | African, South Asian, Chinese | Focus groups | English, Hindi, Urdu, Cantonese, Mandarin, Nepalese, Amharic, Somali, Tigrinya | - Frequent snacking (3) |
| Verrips et al. 1994 ^37^ | “To assess correlates of toothbrushing and toothpaste in four ethnic groups. A further aim was to evaluate the putative role of correlates as intervening variables between ethnicity and caries risk factors.” | Netherlands | 476 parents | Turkish, Moroccan, Dutch, Surinamese | Surveys, Interviews | Turkish, Dutch, Arabic, English | - Awareness of importance of toothbrushing (1) |
| Steckson-Blicks et al. 2008 ^38^ | “To compare data  on caries prevalence and background factors in  4-year-old children from 2007 with data collected  periodically between 1967 and 2002 with the same methods and criteria, and to compare immigrant and non-immigrant children concerning caries  prevalence and background factors.” | Sweden | 218 children including immigrant backgrounds and their parents | Immigrant children | Questionnaires | Not mentioned | - Toothbrushing once a day (3) |

**Table 4. Key findings about oral health related social practices**

| **Social practice** | **Elements of practice - Materials** | **Elements of practice - Meanings** | **Elements of practice - Competences** | **Temporal aspects** | **Spatial aspects** |
| --- | --- | --- | --- | --- | --- |
| Feeding practice bundle | Various kinds of foods and drinks were reported in the included studies: milk and milk products, baby’s formula, ^5,6,14,18,28^ sugar, ^12,32^ sugar and salt containing processed foods (candies, cakes, sweets, biscuits, ice cream, buns, cookies, marmalade/jams, sweet soups, dried fruit, sweetened flakes), ^3,6,12-14,21,35,38^grain-based products containing sugar, ^5,6,15^ solid foods, pureed fruits and veggies ^518^ fresh fruits, ^5,6,15,35^ food additives including traditional ^6,14^ and sweet tasting drinks (vitamin C, fruit juices, and carbonated beverages) ^3-6,13-15,18,27,28,31,35,38^  Indirect materials:  food stores ^35^ television, walker, balloons, toys, books ^14^ utensils, bottles, feeders, ^13^ tea shops, advertisements, ^18^ cups ^20^ | A combination of cultural, emotional and socially shared meanings: maternal preference and anxiety, ^614^ parental feeding beliefs, children’s preference, family feeding expectation, ^14^ parental distress and emotional need, ^31^ peer pressure on dietary choices, ^18^ social expectations ^35^  **giving sweet drinks**: maternal beliefs, health benefits of drinks ^5,14,27^ misconceptions about sweetened drinks, ^28^ preference of sweet taste, ^15^ concerns over water safety from birth country ^18,27^  **giving snacks**: reward behaviour, ^3,32^ calm fussiness, ^5^ social expectations, family and peer pressure, caring norms ^3^  **breastfeeding:** parental preference, antenatal advice, ^28^ cultural norm, birth control, economical^35^  **bottle-feeding:** Convenience, maternal anxiety, ^14^ breastfeeding challenges and experiences, ^28^ advertising influence, ^18^ parental preference for uninterrupted sleep, ^3^ working caregivers’ convenience feeding arrangements, ^15^ social and family expectations ^14,27^ | Caregivers modified diets as per children’s taste. ^4,6,8^ Caregivers used strategies to feed children including distract using tv/balloons when feeding, modifying nipples on bottles to feed large amount of foods, use bottles to measure food intake, ^14^ prechew baby’s food, ^18,24^ sweeten baby’s food, ^28^ read cues from children, ^5,35^ knowledge of recommendations of solid foods, ^6,14,18^ to limit sweet drinks consumption, ^3^ follow western kids’ habits, replace tap water with sweet drinks, ^18^ interpret cues for hydration in children ^5^  When giving snacks it was observed that caregivers knew to limit snack (sweet foods/drinks) consumption ^32,35^ | CALD group of mothers from one study changed feeding patterns throughout the four to 12 months period, ^28^ premastication practice for about six months of age, ^18^ snacks were provided to children as young as one year. ^21^ Snacking processed foods occurred every day to occasionally, several times in a day and in some cases ad libitum ^6,32^  Bottle feeding including night feeding ranged from 15 months up to 4 years ^2,4,5,15,21,24,28,35^  Breastfeeding ranged between under 6 months to up to 2 years, and when in camps breastfed occurred for up to 2 years. ^5,18^ Frequency of drinking sweetened drinks varied from daily to occasionally, in some cases, children drank sweetened drinks through the night. ^5,17,18,22,38^ | Limited access to foods while in camps, ^5^ Changes in diet since moving to host country, ^13^ parental preference to provide all foods available in host country, foods in supermarkets, ^18^ homes and relative’s houses influenced by social visits ^35^ availability of various foods in host country, Accessibility and availability of shops selling sweetened drinks, influence by availability and past experiences of water in home countries ^18^ |
| Using a comforter | Dummies, pacifiers, ^21,33^ bottles, ^5^ sweetening and flavouring agents ^10^ | Distressed child, ^6^ child’s personal preference ^10^ | To coat dummies with sweet flavouring agents, ^6^ what to add in the bottles, ^6^ putting the dummies in their (mothers) own mouth first and then giving it to the baby ^33^ | Use of comforter ranged from night time to frequently during the day. ^6,21^ Children as old as two were provided with comforters ^21^ |  |
| Sleeping | Materials which aid in sleeping such as bottles were reported in the studies. Bottle, ^5,19,35^ cup ^35^ milk/fruit juice, ^5,6,10^ sugary flavoured drinks, ^19,31^ sweetened substances, sweetened pacifiers, breast milk, ^18,33,35^ formula ^18^ | Meanings attached to sleeping were: a well-slept baby is a healthy baby, ^3^ expectation of the baby sleeping well in order for the father needing long rest for long hours in the field (work) ^15^ these meanings led to caregivers providing sweetened drinks in bottles to encourage sleeping. | Bottles were used to put the baby to sleep, ^3,20^ Flavouring agents were used to modify the milk taste,^18,19^ to provide breast/bottle when child woke up, or to aid in sleep, use bottle propping to aid in sleeping ^35^ | Children were given sugary drinks in bottle at 12 months to aid in sleeping, ^21^ mothers provided children with bottle/breast whenever the children woke up and in the night time for sleeping ^35^ | Caregivers provided breast milk with formula in a bottle for sleeping which they were aware of from home country ^18^ |
| Teeth cleaning | Conventional materials: Toothbrush, toothpaste ^4,18,19^ dental floss, towelette ^3^  Non-conventional materials: *Sakra,* ^4^ water, ^3,14^ fingers, cloth, twigs (Neem tree), Indian proprietary tooth powder, homemade mix of salt and burnt breadcrumbs, ^26^ salt, charcoal, betel nut and rocks, *miswak* (sticks), green skin from walnut fruit or nuts ^18^ | Brushing was performed to prevent tooth decay, ^3,16,32,34^ strong and clean teeth, ^19,37^ subjective norms and behavioural control ^16^ | Caregivers knew to assist children with their toothbrushing using toothbrush and toothpaste, ^4,12^ to know the role of fluoridated toothpaste in preventing tooth decay, ^19^ able to circumvent any difficulties that may occur during the child’s toothbrushing performance, ^16^ find motivating ways to make children brush, ^35^  ask husbands to help with child’s toothbrushing, children learning to brush by observing their mother s brushing, ^3^ children to brush, ^34^ to check children’s teeth regarding brushing ^37^ | children brushed twice daily, ^22,35,38^ brushing started before year 1 and ranged between 1 and 3, ^4,32,35^, starting brushing when first tooth appears, ^16^ brushing when woke up, after breakfast, brush again before leaving for school, after returning from school and before bedtime, ^3^ children taught how to brush from a very young age ^34^ | Day care centres taught children how to brush their teeth ^34^ |
| Health and care-oriented mobility bundle | schools, ^18^ medical centres, ^11,13,28^ community centres, day care, federally funded Women Infant and Children (WIC) nutrition sites, prenatal classes ^7,9,34,35^  transportation infrastructure, ^13^ affordability: money, insurance, dental benefits and health care card, ^9,13^ | Trust in their health care providers for advice regarding children, providing healthy meals because of school policies ^11,18^  Visiting health centres when the child was in pain, ^7,9,14,15,35^ encouragement from the community-based resources, ^13^ social and cultural expectations of physical attractiveness, ^18,34^ cultural expectation to listen to dentist rather than parents, ^18^ peer influence to visit the dentist, to have clean teeth, self-esteem, ^3,7^ child care centre policies, ^34^ older siblings’ personal dental experiences ^7^ | Awareness of help at the centres, ^13^ aware of recommendations from healthcare providers regarding children’s oral health and feeding, ^11,28^ Awareness of dental benefit schemes for children, ^4,13^ extended family members booking dental appointments for children, ^14^ to know to visit a dental clinic when noticing a dental problem, ^17^ personal networks influencing dental visits, reading health related books to visits the dental clinics, ^35^ able to drive to the dental clinic ^13^ | Taking a child to the dentist ranged from 2 to 4 years of age or until the child attends elementary school ^7,17,35^ some caregivers only took children when they noticed a dental problem ^18^ Some caregivers waited for becoming eligible to receive publicly funded dental services, No time to take the children to dental clinics because of both parents working multiple jobs and full time, ^13^ Very few dental clinics took children under the age of four ^13^ | Inspections of lunchboxes in schools, ^18^ inconvenient locations of dental clinics, ^15^ lack of transportation to travel to far located clinics ^36^  taking children to home countries for cheap and affordable dental treatment ^13^ |

**References**

1. Bank TW. World Bank Country and Lending Groups. World Bank Group. Accessed 03 May, 2023. <https://datahelpdesk.worldbank.org/knowledgebase/articles/906519-world-bank-country-and-lending-groups>

2. Dykes J, Watt RG, Nazroo J. Socio-economic and ethnic influences on infant feeding practices related to oral health. *Community Dent Health*. 2002;19(3):137-143.

3. Finlayson TL, Beltran NY, Becerra K. Psychosocial factors and oral health practices of preschool-aged children: A qualitative study with Hispanic mothers. *Ethn Health*. 2019;24(1):94-112. doi:<https://dx.doi.org/10.1080/13557858.2017.1315366>

4. Godson JH, Williams SA. Oral health and health related behaviours among three-year-old children born to first and second generation Pakistani mothers in Bradford, UK. *Community Dent Health*. 1996;13(1):27-33.

5. Harrison R, Wong T, Ewan C, Contreras B, Phung Y. Feeding practices and dental caries in an urban Canadian population of Vietnamese preschool children. *Journal of Dentistry for Children*. 1997;64(2):112-117.

6. Hocking BM, Campbell MJA, Storey E. Infant feeding patterns. *Australian Dental Journal*. 1982;27(5):300-305. doi:10.1111/j.1834-7819.1982.tb05251.x

7. Hoeft KS, Barker JC, Masterson EE. Maternal Beliefs and Motivations for First Dental Visit by Low-income Mexican American Children in California. *Pediatric Dentistry*. 2011;33(5):392-398.

8. Contento IR, Basch C, Shea S, et al. Relationship to mothers' food choice criteria to food intake of preschool children: Identification of family subgroups. *Health Education Quarterly*. 1993;20(2):243-259. doi:<https://dx.doi.org/10.1177/109019819302000215>

9. Christian B, Young D, Gibbs L, et al. Exploring child dental service use among migrant families in metropolitan Melbourne, Australia. *Australian Dental Journal*. 2015;60(2):200-4. doi:<https://dx.doi.org/10.1111/adj.12321>

10. Chomitz VR, Park HJ, Koch‐Weser S, et al. Modifying dietary risk behaviors to prevent obesity and dental caries in very young children: results of the Baby Steps to Health pediatric dental pilot. *Journal of Public Health Dentistry*. 2019;79(3):207-214.

11. Hilton IV, Stephen S, Barker JC, Weintraub JA. Cultural factors and children's oral health care: a qualitative study of carers of young children. *Community Dentistry and Oral Epidemiology*. 2007;35(6):429-38.

12. Gibbs L, de Silva AM, Christian B, et al. Child oral health in migrant families: A cross-sectional study of caries in 1-4 year old children from migrant backgrounds residing in Melbourne, Australia. *Community Dent Health*. 2016;33(2):100-106. doi:10.1922/CDH_3698Gibbs07

13. Barker JC, Horton SB. An ethnographic study of Latino preschool children's oral health in rural California: Intersections among family, community, provider and regulatory sectors. *BMC Oral Health*. 2008;8(1 C7 - 8)doi:10.1186/1472-6831-8-8

14. Karasz A, Patel V, Ranasinghe S, Chaudhuri K, McKee D. Preventing caries in young children of immigrant Bangladeshi families in New York: perspectives of mothers and paediatricians. *Community Dent Health*. 2014;31(2):80-4.

15. Lukes SM. Oral health knowledge attitudes and behaviors of migrant preschooler parents. *Journal of dental hygiene : JDH*. 2010;84(2):87-93.

16. Mustafa M, Nasir EF, Anne Nordrehaug Å. Attitudes toward brushing children's teeth—A study among parents with immigrant status in Norway. *International Journal of Paediatric Dentistry*. 2021;31(1):80-88. doi:<https://doi.org/10.1111/ipd.12683>

17. Nguyen UN, Rowe DJ, Barker JC. Survey of Knowledge, Beliefs, and Behaviors of Migrant Vietnamese Parents Regarding Young Children's Oral Health. *Journal of Dental Hygiene*. 2017;91(2):45-53.

18. Nicol P, Al-Hanbali A, King N, Slack-Smith L, Cherian S. Informing a culturally appropriate approach to oral health and dental care for pre-school refugee children: a community participatory study. *BMC Oral Health*. 2014;14:69. doi:<https://dx.doi.org/10.1186/1472-6831-14-69>

19. Watson M-R, Horowitz AM, Carcia I, Canto MT. Caries conditions among 2--5-year-old immigrant Latino children related to parents' oral health knowledge, opinions and practices. *Community Dentistry & Oral Epidemiology*. 1999;27(1):8-15.

20. Weinstein P, Oberg D, Domoto PK, Jeffcott E, Leroux B. A prospective study of the feeding and brushing practices of WIC mothers: Six- and twelve-month data and ethnicity and familial variables. *Journal of Dentistry for Children*. 1996;63(2):113-117.

21. Wendt LK, Birkhed D. Dietary habits related to caries development and immigrant status in infants and toddlers living in Sweden. *Acta Odontologica Scandinavica*. 1995;53(6):339-344. doi:10.3109/00016359509005998

22. Stecksén-Blicks C, Sunnegårdh K, Borssén E. Caries Experience and Background Factors in 4-Year-Old Children: Time Trends 1967-2002. *Caries Research*. 2004;38(2):149-155.

23. Verrips GH, Frencken JE, Kalsbeek H, ter Horst G, Filedt Kok‐Weimar TL. Risk indicators and potential risk factors for caries in 5‐year‐olds of different ethnic groups in Amsterdam. *Community Dentistry and Oral Epidemiology*. 1992;20(5):256-260. doi:10.1111/j.1600-0528.1992.tb01694.x

24. Werneck RI, Lawrence HP, Kulkarni GV, Locker D. Early Childhood Caries and Access to Dental Care among Children of Portuguese-Speaking Immigrants in the City of Toronto. *Journal of the Canadian Dental Association*. 2008;74(9):805-805.

25. Wendt L-K, Hallonsten A-L, Koch G, Birkhed D. Oral hygiene in relation to caries development and immigrant status in infants and toddlers. *Scandinavian journal of dental research*. 1994;102(5):269-273.

26. Williams SA, Fairpo CG. Cultural variations in oral hygiene practices among infants resident in an inner city area. *Community Dent Health*. 1988;5(3):265-271.

27. Williams SA, Sahota P. An enquiry into the attitudes of Muslim Asian mothers regarding infant feeding practices and dental health. *Journal of Human Nutrition and Dietetics*. 1990;3(6):393-401. doi:10.1111/j.1365-277X.1990.tb00249.x

28. Williams SA, Sahota P, Fairpo CG. Infant feeding fractices within white and Asian Communities in inner‐city Leeds. *Journal of Human Nutrition and Dietetics*. 1989;2(5):325-338. doi:10.1111/j.1365-277X.1989.tb00035.x

29. Skeie MS, Espelid I, Riordan PJ, Klock KS. Caries increment in children aged 3-5 years in relation to parents' dental attitudes: Oslo, Norway 2002 to 2004. *Community Dentistry and Oral Epidemiology*. 2008;36(5):441-450. doi:10.1111/j.1600-0528.2008.00430.x

30. Skeie MS, Klock KS, Haugejorden O, Riordan PJ, Espelid I. Tracking of parents' attitudes to their children's oral health-related behavior-Oslo, Norway, 2002-04. *Acta Odontologica Scandinavica*. 2010;68(1):49-56. doi:10.3109/00016350903364926

31. Skeie MS, Riordan PJ, Klock KS, Espelid I. Parental risk attitudes and caries-related behaviours among immigrant and western native children in Oslo. *Community Dentistry and Oral Epidemiology*. 2006;34(2):103-13.

32. Skaret E, Espelid I, Skeie MS, Haugejorden O. Parental beliefs and attitudes towards child caries prevention: Assessing consistency and validity in a longitudinal design. *BMC Oral Health*. 2008;8(1 C7 - 1)doi:10.1186/1472-6831-8-1

33. Shiboski CH, Gansky SA, Ramos-Gomez F, Ngo L, Isman R, Pollick HF. The association of early childhood caries and race/ethnicity among California preschool children. *Journal of Public Health Dentistry*. 2003;63(1):38-46. doi:10.1111/j.1752-7325.2003.tb03472.x

34. Rivera Y, Boyd LD, Libby L. Hispanic Seasonal Farmworker Caregivers' Beliefs and Perceptions of Early Childhood Caries. *Journal of Dental Hygiene*. 2020;94(5):14-21.

35. Riedy CA, Weinstein P, Milgrom P, Bruss M. An ethnographic study for understanding children's oral health in a multicultural community. *International Dental Journal*. 2001;51(4):305-312. doi:10.1002/j.1875-595X.2001.tb00843.x

36. Perez A, Amin M. Dimensional analysis of psychosocial barriers to prevention of early childhood caries among recent immigrants. *SAGE Open*. 2014;4(2)doi:10.1177/2158244014539331

37. Verrips GH, Kalsbeek H, Van Woerkum CM, Koelen M, Kok-Weimar TL. Correlates of toothbrushing in preschool children by their parents in four ethnic groups in The Netherlands. *Community Dent Health*. 1994;11(4):233-9.

38. Stecksén-Blicks C, Kieri C, Nyman J, Pilebro C, Borssén E. Caries prevalence and background factors in Swedish 4-year-old children -- a 40-year perspective. *International Journal of Paediatric Dentistry*. 2008;18(5):317-324. doi:10.1111/j.1365-263x.2008.00929.x
